# Increased COUP-TFII expression in adult hearts induces mitochondrial dysfunction resulting in heart failure

**DOI:** 10.1038/ncomms9245

**Published:** 2015-09-10

**Authors:** San-Pin Wu, Chung-Yang Kao, Leiming Wang, Chad J. Creighton, Jin Yang, Taraka R. Donti, Romain Harmancey, Hernan G. Vasquez, Brett H. Graham, Hugo J. Bellen, Heinrich Taegtmeyer, Ching-Pin Chang, Ming-Jer Tsai, Sophia Y. Tsai

**Affiliations:** 1 Department of Molecular and Cellular Biology, Baylor College of Medicine, Houston, Texas 77030, USA; 2 Adrienne Helis Malvin Medical Research Foundation, New Orleans, Louisiana 70130, USA; 3 Department of Medicine, Baylor College of Medicine, Houston, Texas 77030, USA; 4 Dan L. Duncan Cancer Center, Baylor College of Medicine, Houston, Texas 77030, USA; 5 Department of Cardiology, Indiana University School of Medicine, Indianapolis, Indiana 46202, USA; 6 Department of Molecular and Human Genetics, Baylor College of Medicine, Houston, Texas 77030, USA; 7 Department of Internal Medicine, University of Texas Medical School at Houston, Houston, Texas 77030, USA; 8 Program in Developmental Biology, Baylor College of Medicine, Houston, Texas 77030, USA

## Abstract

Mitochondrial dysfunction and metabolic remodelling are pivotal in the development of cardiomyopathy. Here, we show that myocardial COUP-TFII overexpression causes heart failure in mice, suggesting a causal effect of elevated COUP-TFII levels on development of dilated cardiomyopathy. COUP-TFII represses genes critical for mitochondrial electron transport chain enzyme activity, oxidative stress detoxification and mitochondrial dynamics, resulting in increased levels of reactive oxygen species and lower rates of oxygen consumption in mitochondria. COUP-TFII also suppresses the metabolic regulator *PGC-1* network and decreases the expression of key glucose and lipid utilization genes, leading to a reduction in both glucose and oleate oxidation in the hearts. These data suggest that COUP-TFII affects mitochondrial function, impairs metabolic remodelling and has a key role in dilated cardiomyopathy. Last, *COUP-TFII* haploinsufficiency attenuates the progression of cardiac dilation and improves survival in a calcineurin transgenic mouse model, indicating that COUP-TFII may serve as a therapeutic target for the treatment of dilated cardiomyopathy.

## Abstract (web summary)

Transcription factor COUP-TFII is elevated in the hearts of non-ischaemic cardiomyopathy patients, but the nature of this correlation is unknown. Here the authors show that forced cardiac expression of COUP-TFII in mice causes dilated cardiomyopathy because of altered mitochondrial function and impaired metabolic remodelling.

## Graphical abstract

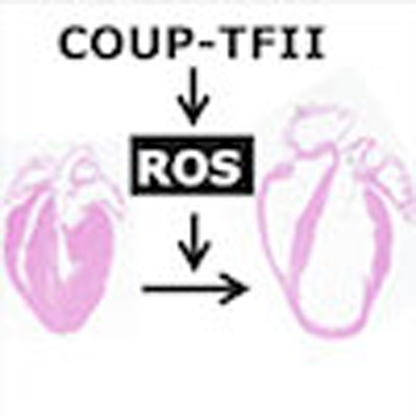

Heart failure is the leading cause of death in developed countries, and dilated cardiomyopathy (DCM) is the most common form of heart failure^1^. The aetiology for the majority of non-ischaemic DCM remains unclear. Studies on human specimens and animal models suggest that impaired mitochondrial electron transport chain (ETC) reduces production of high-energy phosphates^2^,^3^,^4^, leading to energy starvation of the cells. Although the mitochondrial ETC primarily produces ATP, it also generates reactive oxygen species (ROS) as part of a normal respiration process^5^. A defective ETC has been linked to excessive production of ROS^6^, which imposes oxidative stress in failing hearts by damaging mitochondrial DNA and proteins and triggering more ROS formation^7^. In addition, mitochondrial dynamics also contribute to mitochondrial homeostasis in the hearts. Impairment of mitochondrial fusion by *Mfn1*/*Mfn2* double knockout (DKO) results in mitochondrial fragmentation, respiratory dysfunction, leading to a rapid development of DCM^8^.

Metabolic remodelling also emerges as a major player in pathogenesis of heart failure. We have proposed that metabolic remodelling precedes, initiates and sustains functional and structural remodelling^9^. The *PGC-1* regulatory network is known as the major network-modulating cardiac metabolism. This network comprises coregulators PGC-1α and PGC-1β that coactivate multiple nuclear receptors, including estrogen-related receptor (ERR)α, ERRγ and peroxisome proliferator-activated receptor (PPAR)α, to control expression of genes essential for energy and mitochondrial homeostasis^10^,^11^,^12^,^13^. Loss of key members in this regulatory network produces a range of metabolic defects, including heart failure, defective mitochondrial biogenesis and dynamics and maladaptation to cardiac stress in mice^10^,^11^,^12^,^13^.

COUP-TFII (Nr2f2), a member of the nuclear receptor family, is highly expressed in the embryonic atria^14^, whereas its expression in ventricular cardiomyocytes remains very low from embryo to adult^14^,^15^. Under pathological conditions, the expression of COUP-TFII is elevated in the stressed ventricles of non-ischaemic cardiomyopathy patients and a pressure overload mouse model^16^,^17^.

In the present study, we generated a mouse model by ectopically expressing COUP-TFII in adult cardiomyocytes to understand the role of COUP-TFII in the development of cardiomyopathy. Increased COUP-TFII levels alter expression of key mitochondrial and metabolic genes, enhance oxidative stress, disturb metabolic homeostasis and lead to DCM. On the other hand, reduced *COUP-TFII* expression partially mitigates calcineurin-induced cardiac dysfunction and improves survival of calcineurin transgenic mice. Our results reveal the causative role of COUP-TFII in the development of heart failure.

## Results

### Increased COUP-TFII expression in stressed hearts

**Figure 1:**
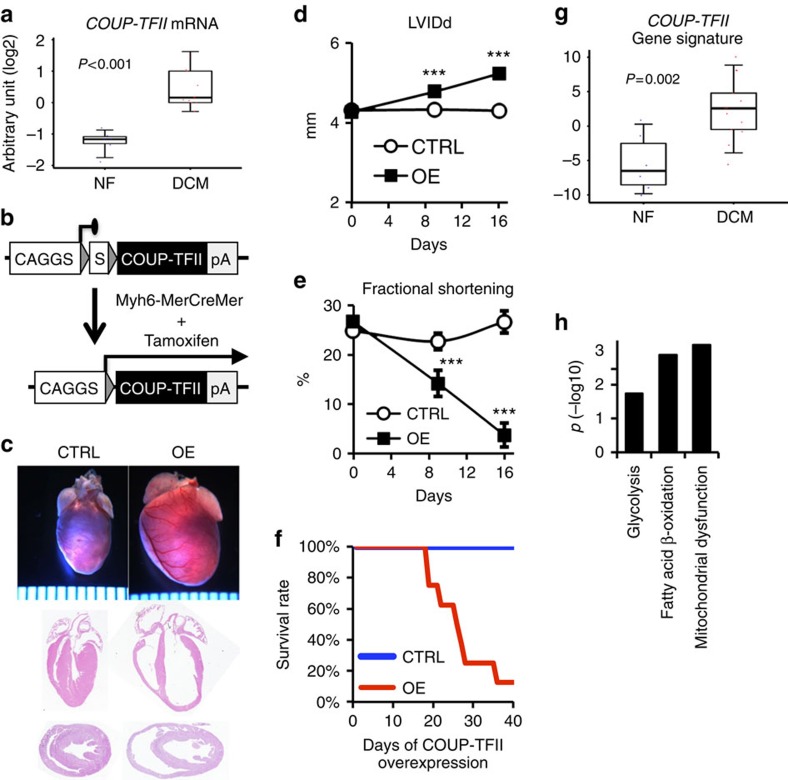
Myocardial COUP-TFII expression causes dilated cardiomyopathy (DCM). (**a**) *COUP-TFII* mRNA levels in human heart tissues of non-failing (NF, *n*=6) and idiopathic DCM (*n*=13) from GSE1869. (**b**) Strategy of inducible COUP-TFII expression in adult mouse myocardium. (**c**) Gross view of day 16 hearts. The scale of rulers underneath the whole mount images is 1 mm. (**d**,**e**) Time-coursed echocardiogram. Day 0 indicates the day before induction of COUP-TFII expression. LVIDd, left ventricular interior dimension at diastole. *N*=5 (CTRL) and 7 (OE). (**f**) Survival rate of nine CTRL and eight OE mice over time. (**g**) Manifestation of the *COUP-TFII* gene signature in human heart samples (GSE1869). *COUP-TFII* gene signature is curated from all COUP-TFII downstream targets in mouse hearts that are identified by the microarray analysis. (**h**) Major biological processes altered by COUP-TFII overexpression identified by the Ingenuity pathway analysis. ***P<0.001 (*t*-test) between CTRL and OE. Error bars denote the standard error of the mean (s.e.m.).

When we reviewed available human DCM data sets, we found a significant increase in *COUP-TFII* expression levels (3.2-fold) in 13 myocardial tissues of end-stage non-ischaemic DCM^16^ (Fig. 1a). In a second cohort of patients, an average of 1.8-fold increase on *COUP-TFII* levels was also observed in the heart of 86 patients with idiopathic DCM (GSE5406)^18^. Results from these two independent cohorts of patients suggest an association between the ventricular *COUP-TFII* levels and DCM in human.

We found that in response to stress imposed by transaortic constriction (TAC), the expression of ventricular *COUP-TFII* mRNA was induced in mice (Supplementary Fig. 1a). This result is consistent with previous findings of increased COUP-TFII protein levels in this model^17^. Similarly, ventricles of *calcineurin* transgenic mice (CnTg), known to develop hypertrophy and subsequent DCM, also exhibited an elevated expression of the *COUP-TFII* gene (Supplementary Fig. 1b). In addition, COUP-TFII protein levels were increased in isolated cardiomyocytes of CnTg mice (Supplementary Fig. 1c). Together, these results implicate a strong association of increased *COUP-TFII* expression with cardiomyopathy in mice and in humans.

### COUP-TFII induces DCM

The potential link to cardiomyopathy prompted us to investigate whether increased COUP-TFII expression in mice might impact the development of contractile dysfunction. For this purpose, we crossed a previously established *COUP-TFII* overexpression allele *CAG-S-COUP-TFII* with a cre driver *Myh6-MerCreMer* (*Myh6-MCM*) line^14^,^19^ to overexpress COUP-TFII specifically in cardiomyocytes after heart development is complete, by administration of tamoxifen (Fig. 1b). Functionally, overexpressing COUP-TFII (OE, *Myh6-MCM; CAG-S-COUP-TFII*) in 2-month-old mice resulted in a greatly enlarged heart in comparison with the CTRL (*Myh6-MCM*) mice (Fig. 1c) 16 days after *COUP-TFII* transgene induction (D16). Echocardiography further revealed that OE mice exhibited characteristics of DCM, including increased left ventricular interior dimension (Fig. 1d and Supplementary Fig. 1e), reduced fractional shortening (Fig. 1e) and decreased relative wall thickness (RWT; Supplementary Fig. 1f, right panel). The progressive compromise of cardiac function resulted in increased mortality of OE mice after activation of COUP-TFII expression (Fig. 1f). Notably, day 16 OE hearts also had a 5.3-fold increase of *COUP-TFII* mRNA levels over CTRL (Supplementary Fig. 1g). By this time, the OE hearts exhibited severe dilation and contractile dysfunction analogous to end-stage DCM in human patients. The overexpressed *COUP-TFII* mRNA levels between human specimens (3.2X, Fig. 1a) and OE hearts (5.3X, Supplementary Fig. 1g) are not too far apart, suggesting that the mouse model is relevant. Most importantly, the activated *COUP-TFII* transgene increases COUP-TFII protein levels in cardiomyocytes (Supplementary Fig. 1d) to a similar extent as CnTg does (Supplementary Fig. 1c), which gives us confidence to use this model for dissecting the role of COUP-TFII in cardiomyopathy. In summary, our animal model shows that increased COUP-TFII expression in the heart is sufficient to induce DCM with a high mortality rate.

At the molecular level, OE ventricles exhibited decreased *αMHC*, increased *βMHC* and reduced *SERCA2a* gene expression (Supplementary Fig. 1h), similar to the molecular changes observed in human DCM patients and other mouse models. To gain insights on COUP-TFII’s effect in hearts, we identified the molecular profile of day 16 OE hearts through microarray analysis using ventricular compartments. Unbiased Gene Ontology analysis of the OE profile showed significant enrichment in the category of ‘DCM’ in the Kyoto Encyclopedia of Genes and Genomes (*P*=2.2 × 10^−4^), further supporting the pivotal role of COUP-TFII in DCM. Moreover, using the OE profile as a transcriptomic signature of COUP-TFII activities, we found that the COUP-TFII signature is considerably higher in the DCM patients than the CTRL group (Fig. 1g). This finding suggests a stronger presence of COUP-TFII activities in DCM hearts as a result of higher levels of COUP-TFII expression (Fig. 1a). Collectively, our data reveal that the COUP-TFII signalling also exists in the human hearts and is positively correlated with a pathological profile of DCM.

Mechanistically, the Ingenuity Pathway analysis reveals ‘mitochondrial dysfunction’, ‘fatty acid β-oxidation’ and ‘glycolysis’ as the most prominent pathways affected by COUP-TFII overexpression in cardiomyocytes (Fig. 1h). This result suggests that COUP-TFII alters cardiac energy homeostasis via modulating expression of genes important for energy production, fuel utilization and mitochondrial function.

### Impaired cardiac fuel utilization

**Figure 2:**
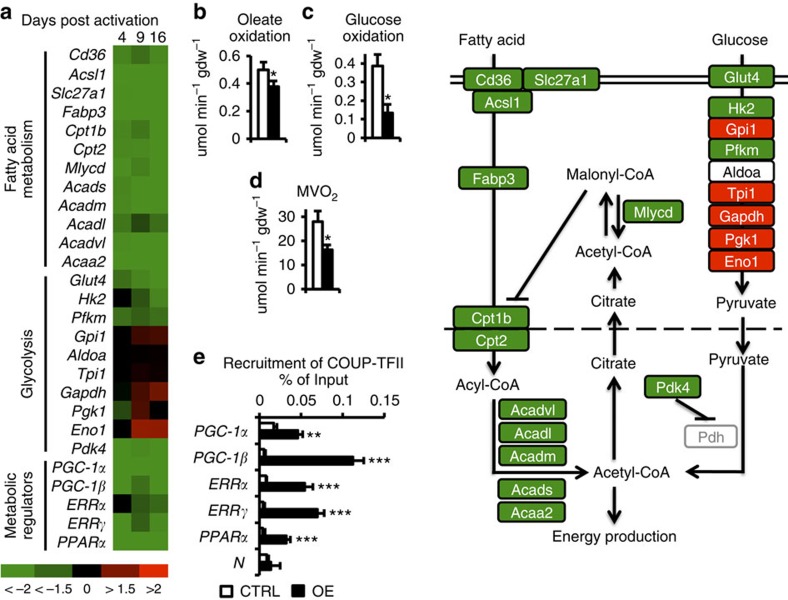
Impaired fuel utilization in COUP-TFII OE ventricles. (**a**) Relative mRNA levels by qRT–PCR in days 4, 9 and 16 ventricles. *N*=5 (CTRL) and 7 (OE). Colour-coded mRNA levels depict relative folds of OE over control hearts. (**b**–**d**) Measurement of oleate oxidation (**b**), glucose oxidation (**c**) and oxygen consumption (**d**) rates in isolated whole hearts at day 9 via Langendorff preparation. *N*=5 (CTRL) and 7 (OE). (**e**) ChIP-qPCR analysis on day 4 hearts. *N*=3 for each genotypes. Diagram on the right depicts fuel utilization genes affected by COUP-TFII OE. Green marks downregulated genes and red labels upregulated genes in response to increased COUP-TFII expression. Expression of genes with the white background is not altered by COUP-TFII. *P<0.05; **P<0.01; ***P<0.001 (*t*-test) between CTRL and OE. Error bars denote the s.e.m.

The OE profile shows a significantly reduced expression of many genes that controls fuel utilization. Quantitative reverse transcription–PCR (qRT–PCR) analysis revealed that genes for uptake, binding, trafficking into the mitochondria and oxidation of fatty acids all exhibited a reduced expression in OE hearts as early as day 4 (Fig. 2a). These findings suggest that fatty acid utilization may be limited in the OE hearts. Indeed, the OE hearts exhibited a lower oleate oxidation rate *ex vivo* (Fig. 2b), as predicted by the molecular profile of the OE hearts.

A comprehensive analysis of the genes implicated in glucose usage of OE hearts showed decreased mRNA levels of *Glut4*, *Hk2* and *Pfkm* (Fig. 2a) genes required for glucose uptake, phosphorylation and subsequent glycolysis, respectively. These findings suggest that OE hearts are also defective in glucose utilization. This was corroborated by the reduced glucose oxidation rates in *ex vivo* OE hearts (Fig. 2c). Interestingly, we also observed an increase in the expression of a subset of genes in the glycolytic pathways (Fig. 2a). However, these changes, which may be a compensatory effect, were not able to offset the impairment in glucose utilization. The reduced fatty acid and glucose oxidation rates are accompanied with decreased oxygen consumption rate (OCR) in the OE hearts (Fig. 2d), suggesting a defect in oxidative phosphorylation for energy production.

The *PGC-1* network is critical in regulating genes essential for cardiac metabolism^10^,^13^. Because the expression of *PGC-1α* is upregulated in the adipose tissue of *COUP-TFII* heterozygous mutant mice^20^, it is likely that *PGC-1α* expression is also altered when COUP-TFII is overexpressed in the hearts. Indeed, *PGC-1α* expression was reduced when COUP-TFII is overexpressed (Fig. 2a). Moreover, levels of other members of the *PGC-1* network, including *PGC-1β*, *ERRα*, *ERRγ* and *PPARα* are all reduced in cardiac tissues in the OE hearts (Fig. 2a). The notion that COUP-TFII suppresses the *PGC-1* network is supported by the inverse relationship of *COUP-TFII* and *PGC-1α/β*^11^, *ERRα*^21^ and *PPARα*^22^ signatures (Supplementary Fig. 2). These results indicate that COUP-TFII is a major metabolic regulator that is central to control cardiac fuel metabolism.

Because *PGC-1α* is a direct downstream target of COUP-TFII in adipocytes^20^, it is likely that the same regulatory mechanism also operates in the heart. Using chromatin immunoprecipitation (ChIP)-quantitative PCR (qPCR) assays, we found that COUP-TFII indeed binds to a previously identified intronic COUP-TFII-binding site in adipocytes (Fig. 2e)^20^. This result confirms that *PGC-1α* is likely a direct target of COUP-TFII in the heart and that the mechanism of action by which COUP-TFII regulates *PGC-1α* is conserved among various cell types. In addition to *PGC-1α*, COUP-TFII also directly binds to *ERRα*, *ERRγ* and *PPARα* genes in ChIP assays (Fig. 2e). Collectively, our results indicate a direct regulatory role of COUP-TFII for these master metabolic regulators.

### Development of mitochondrial dysfunction

**Figure 3:**
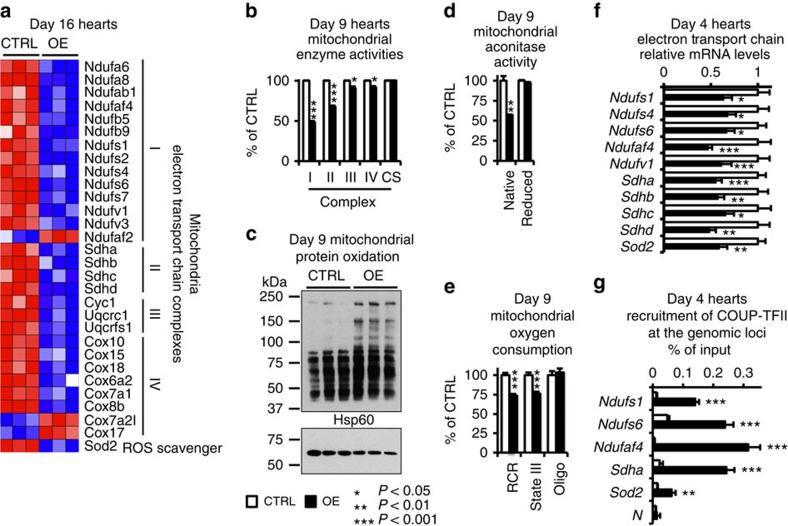
Increased ROS production and compromised mitochondria functions. (**a**) Expression profile of affected mitochondrial genes in day 16 hearts. Blue marks downregulated and red depicts upregulated genes. (**b**–**e**) Day 9 hearts. (**b**) Activities of mitochondrial ETC enzymes normalized to citrate synthase (CS) activities. (**c**) Oxyblot analysis on mitochondria protein oxidation status. Hsp60 serves as loading control. (**d**) Activities of mitochondria aconitase at native and reduced forms, normalized to total mitochondria protein levels. *N*=3 for each genotype. (**e**) Mitochondria oxygen consumption rates. (**f**,**g**) Day 4 hearts. (**f**) Expression levels of key mitochondria ETC and ROS scavenger genes. *N*=6 (CTRL) and 10 (OE). (**g**) ChIP-qPCR results of COUP-TFII binding at genomic loci of ETC and ROS scavenger genes. *N*=3 for each genotype. RCR, respiratory control ratio. Oligo, effect of oligomycin. *N*=3 for each genotypes. *P<0.05; **P<0.01; ***P<0.001 (*t*-test) between CTRL and OE. Error bars denote the s.e.m.

We next examine whether mitochondria are dysfunctional in the OE hearts, based on downregulation of the expression of many genes that encode components in complexes I, II, III and IV of the mitochondrial ETC (Fig. 3a). Transcript analysis further confirmed suppression of ETC gene expression 9 days post induction of COUP-TFII expression when the OE hearts are at the early phase of heart failure (Supplementary Fig. 3a). The effect of altered ETC gene expression is manifested in the reduction of enzyme activities of complexes I, II, III and IV in isolated mitochondria of OE ventricles (Fig. 3b). This defect on ETC enzyme complexes may generate excessive production of ROS and increase oxidative stress. In addition, decreased expression of mitochondrial ROS scavenger gene *Sod2* in the OE hearts (Fig. 3a and Supplementary Fig. 3a) may reduce the ROS detoxifying capacity and contribute to an increase in ROS levels. In line with this gene expression profile, higher levels of oxidized proteins were observed in mitochondria of day 9 OE hearts in the Oxyblot assay (Fig. 3c). As a result, mitochondrial protein function is compromised in the OE hearts, as reflected in the reduction of mitochondrial aconitase activity, which is highly sensitive to oxidative stress (Fig. 3d). Control experiments carried out in a reduced condition reveal that, without the impact of oxidation, total aconitase activities are comparable between CTRL and OE mice, suggesting excessive oxidation as the primary cause of reduction in enzyme activities. The consequence of heightened oxidative stress is manifested in a decreased mitochondrial OCR (Fig. 3e) and a lower mitochondrial ATP content (Supplementary Fig. 3b) suggesting a reduction in oxidative phosphorylation capacity and ATP production in mitochondria of the day 9 OE hearts. Notably, the number and density of mitochondria are comparable between OE and CTRL hearts, as indicated by the similar mitochondria DNA content and volume density (Supplementary Fig. 3c,d, respectively). It is not surprising that mitochondrial number and density are not altered in response to decreased *PGC-1α* and *PGC-1β* levels, because Martin *et al.* already reported similar observations in adult *PGC-1α/β* DKO hearts^11^. Perhaps the rate of mitochondria biogenesis slows down at the adult stage, rendering the effect of losing *PGC-1α/β* less pronounced as compared with the perinatal period where mitochondria rapidly propagate. Thus, our findings indicate that decreased ATP production in mitochondria because of increased oxidative stress, rather than the change of mitochondrial numbers, is one of the major contributors of the defective energy metabolism observed in the OE heart.

To confirm that the observed alterations of the mitochondrial metabolism in the heart are caused by COUP-TFII overexpression and are not secondary to heart failure, we examine the expression of COUP-TFII target genes in earlier time point before onset of heart failure. Day 4 is likely the earliest time point for induction of COUP-TFII expression subsequent to 3-day treatment of tamoxifen before the exhibition of defects in the heart function. As anticipated, echocardiogram results indeed show no significant difference in ventricular chamber size, wall thickness and ejection fraction between CTRL and OE hearts (Supplementary Fig. 3e–g), indicating that day 4 OE hearts are structurally and functionally comparable to CTRLs. Results of qRT–PCR analyses on day 4 hearts reaffirm that suppression of gene expression by COUP-TFII precedes the development of gross differences between genotypes (Fig. 3f). Moreover, we also find enhanced recruitment of COUP-TFII to the genomic loci of *Ndufs1*, *Ndufs6*, *Ndufaf4*, *Sdha* and *Sod2* genes by ChIP assays in the day 4 OE hearts (Fig. 3g), corroborating the notion that COUP-TFII is likely to directly regulate the expression of these genes. Importantly, the mitochondrial respiration rate is also lower in day 4 OE hearts than that of CTRLs (Supplementary Fig. 3h), consistent with the COUP-TFII-dependent changes of gene expression. Taken together, our data demonstrate that regulation of mitochondrial gene expression and function by COUP-TFII occurs at the time when OE hearts are structurally and functionally similar to the CTRL hearts. These findings are indicative of the facts that dysregulation of mitochondrial function in the OE mice is a result of COUP-TFII signalling rather than a secondary consequence of heart failure.

**Figure 4:**
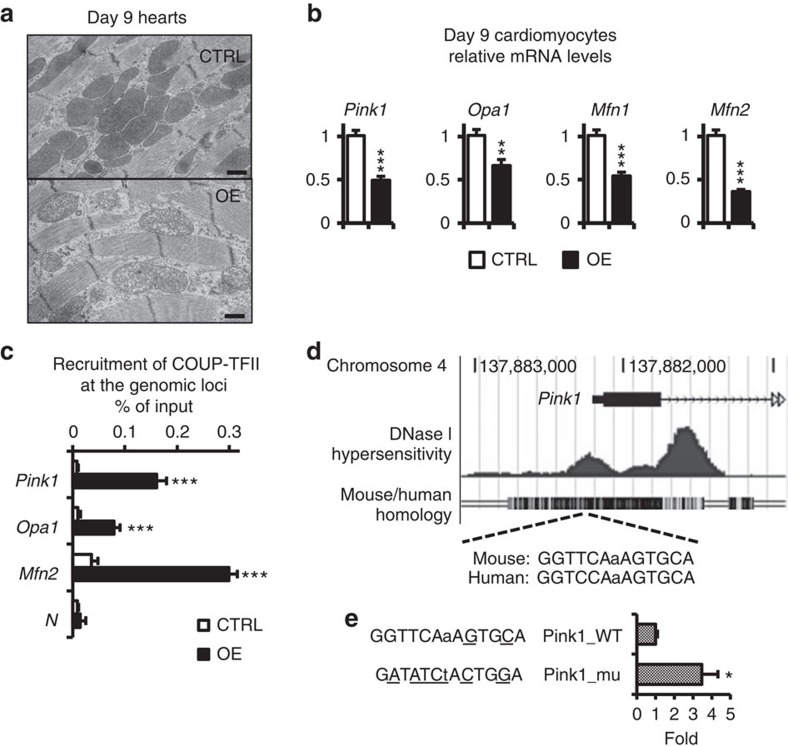
Defects in mitochondrial dynamics. (**a**) Representative electron micrographs of day 9 ventricles from denoted genotypes. Scale bars, 0.5 μm. (**b**) Relative mRNA levels of major mitochondria dynamics genes in day 9 isolated cardiomyocytes. *N*=3 for each genotypes. (**c**) ChIP-qPCR results of COUP-TFII binding at genomic loci of mitochondria dynamic genes. *N*=3 for each genotype. (**d**) Diagram of an enriched COUP-TFII-binding region at the mouse *Pink1* locus identified by ChIP-qPCR shown in **c**, in which DNaseI hypersensitivity result of 8-week-old hearts (from UCSC genome browser, mm9), and a conserved COUP-TFII-binding sequence are shown. (**e**) Luciferase reporter analysis illustrates relative activities of wild-type (WT) and mutant (mu) COUP-TFII-binding sites in C2C12 cells. *P<0.05; **P<0.01; ***P<0.001 (*t*-Test) between CTRL and OE. Error bars denote the s.e.m.

Interestingly, we found that mitochondria of day 9 OE hearts exhibit a roundish shape with reduced electron density and increased distance between cristae (Fig. 4a), suggesting an accumulation of unhealthy mitochondria. Consistent with the morphological findings, we observed a reduction in the expression of key mitochondrial dynamics genes in OE hearts, including *Pink1* for removal of damaged mitochondria and *Opa1*, *Mfn1* and *Mfn2* for fusion and regeneration of healthy mitochondria (Fig. 4b and Supplementary Fig. 4a), whereas no changes were seen in the expression of fission genes *Fis1*, *Mff* and *Dnm1l* (Supplementary Fig. 4a). Furthermore, protein levels of Pink1, Mfn1 and Mfn2 were reduced in isolated cardiomyocytes of OE hearts at the early failing stage (day 9, Supplementary Fig. 4b), indicating that COUP-TFII may directly control the expression of these genes. The reduced expression of *Pink1* and *Mfn2*, two of three major members of the mitochondrial quality control pathway^23^,^24^, in the OE hearts may disrupt the mitochondrial dynamics and contribute to the accumulation of damaged mitochondria as seen in the electron micrograph (Fig. 4a). Collectively, these findings suggest that increased COUP-TFII perturbs mitochondrial homeostasis through suppressing the expression of mitochondrial dynamics genes.

Results from ChIP-qPCR assays further reveal *in vivo* binding of COUP-TFII on genomic loci of the mitochondrial dynamics genes *Pink1*, *Opa1* and *Mfn2* of adult hearts (Fig. 4c), suggesting that these genes are direct targets of COUP-TFII. Next, we used a conserved COUP-TFII-binding site of the *Pink1* gene as an example to demonstrate direct regulation of *Pink1* by COUP-TFII through these *cis*-acting elements. This COUP-TFII-binding site is located between 93 and 105 base pairs upstream of the transcription start site, within a stretch of highly conserved nucleotides between human and mouse. This site is surrounded by open chromatin as evident by the high DNaseI sensitivity (Fig. 4d). A stretch of conserved nucleotides from this region that contain the wild-type (Pink1_WT) or mutant (Pink1_mu) COUP-TFII-binding sites was cloned in front of a TATA-promoter-driven luciferase reporter for luciferase assay in C2C12 cells. The COUP-TFII-binding site shows reduced reporter activities compared with that of the mutant site (Fig. 4e), suggesting a repressive role in the regulation of *Pink1* gene expression. Taken together, the results indicate that binding of COUP-TFII at genomic loci represses expression of mitochondrial dynamics genes.

### The COUP-TFII regulatory network in human

**Figure 5:**
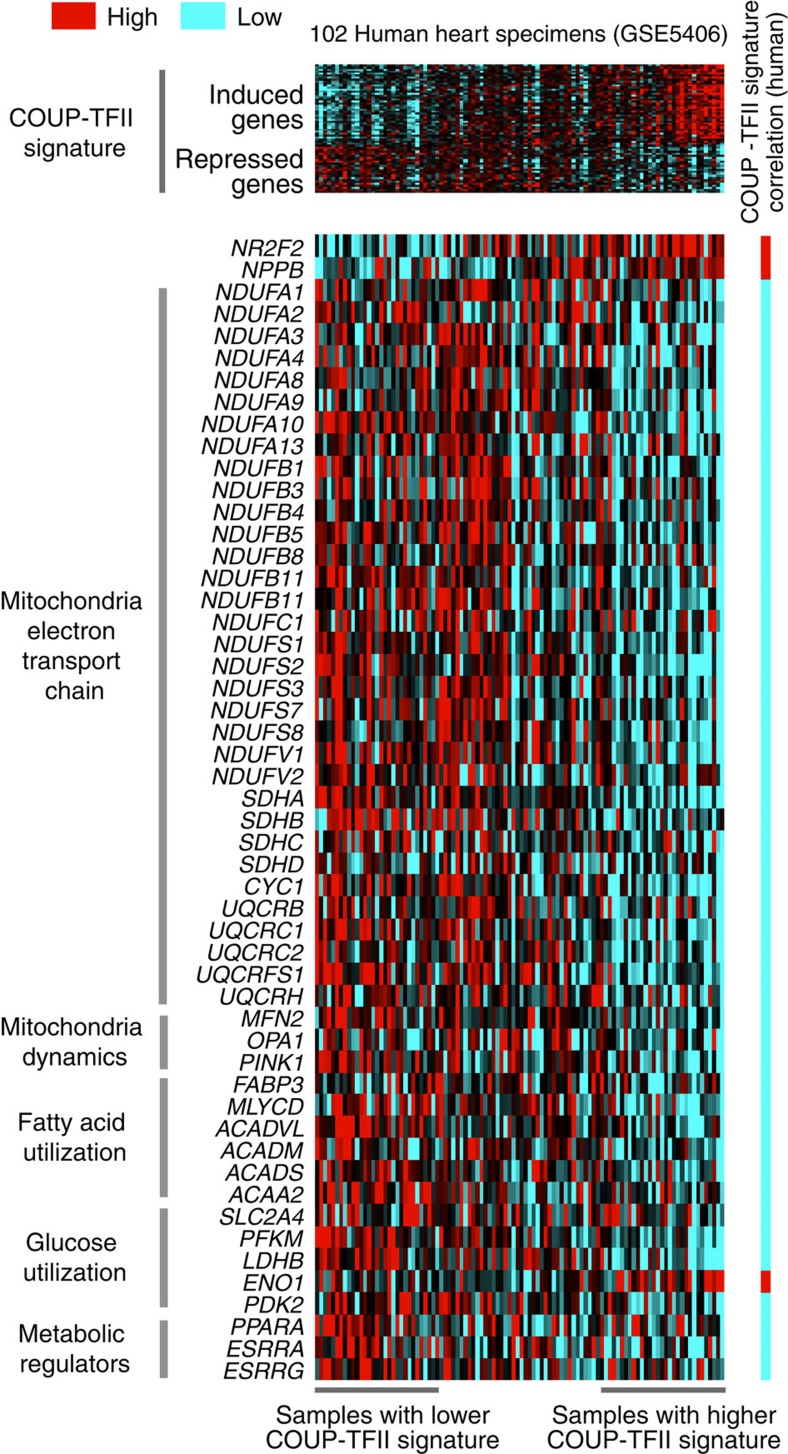
A COUP-TFII-dependent regulatory network in human heart samples. (Top panel) Expression array data of 86 failing and 16 non-failing human left ventricular myocardium from GSE5406, for genes in a transcriptional signature of COUP-TFII, with samples ordered based on manifestation of the signature. (Bottom panel) Heat map shows levels of genes of interest in individual samples corresponding to the order in the top panel. Direction and statistical significance of correlation between expression of genes of interest and COUP-TFII signature are noted at side. Red denotes positive correlation (*P*<0.05, Pearson’s) and blue denotes negative correlation.

The pivotal role of COUP-TFII in regulation of mitochondrial and metabolic genes observed in mice, here, also occurs in human, as supported by publicly available data from patient specimens (Fig. 5). COUP-TFII activity was scored and ranked in RNA profiles of 102 human ventricles (GSE5406) based on the mouse *COUP-TFII* gene signature (Fig. 5, top panel). A higher *COUP-TFII* signature indicates a stronger COUP-TFII activity in a sample. We first observed a positive and significant correlation between COUP-TFII levels and activities in these human samples (Fig. 5). This result supports the notion that the COUP-TFII regulatory network identified in mice also operates in the human heart. In addition, there is a positive correlation between COUP-TFII activity and levels of a cardiac stress marker *Nppb* (Fig. 5), suggesting that increased COUP-TFII signalling may be associated with cardiac stress in human hearts, consistent with what we observed in multiple mouse models.

By comparing COUP-TFII activities and the expression levels of genes of interest, we found significant and inverse correlations between COUP-TFII activities and levels of many mitochondrial genes that encode ETC enzymes, including 23 complex I, 4 complex II and 6 complex III genes (Fig. 5). Moreover, levels of mitochondrial dynamics genes *MFN2, OPA1* and *PINK1* are also negatively correlated with COUP-TFII activities (Fig. 5). These findings are consistent with an essential role of COUP-TFII in suppression of mitochondrial functions and homeostasis in patients.

Similarly, inverse correlations were found between COUP-TFII activities and levels of energy metabolic genes, including fatty acid catabolic genes *FABP3, MLYCD, ACADVL, ACADM, ACADS* and *ACAA2*, glucose metabolic genes *SLC2A4,* *PFKM, LDHB* and *PDK2*, and major metabolic regulators *ERRα, ERRγ* and *PPARα* (Fig. 5). The compensatory effect of increasing expression of the glycolytic gene *ENO1* is also observed in the human heart (Fig. 5), further supporting the presence of a conserved regulatory mechanism for cardiac metabolism. In summary, expression of energy metabolism genes is suppressed when *COUP-TFII* levels are elevated in the human hearts.

### COUP-TFII reduction attenuates disease progression

**Figure 6:**
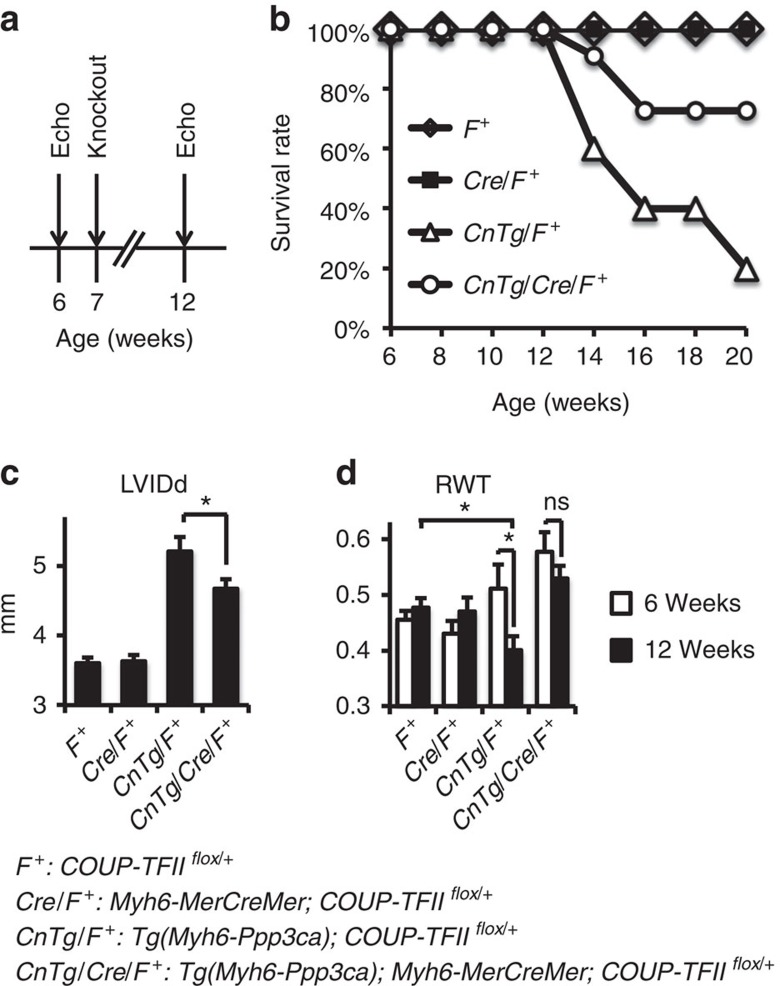
*COUP-TFII* haploinsufficiency partially rescues *calcineurin* transgenic mice. (**a**) Experimental design. Echo, echocardiography. (**b**) Kaplan–Meier curve of mice with denoted genotypes over a 20-week observation period. (**c**) Left ventricular interior dimension at diastole at indicated time points. (**d**) Left ventricle relative wall thickness. Genotypes are denoted at bottom. *N*=10 (F^+^), 10 (*Cre/F*^*+*^), 5 (*CnTg/F*^*+*^) and 11 (*CnTg/Cre/F*^*+*^). *P<0.05; NS, *P*>0.05 (*t*-test) between denoted groups. Error bars denote the s.e.m.

The *calcineurin* transgenic mice develop DCM^25^ and expression of COUP-TFII is elevated in these mice (Supplementary Fig. 1b,c). We therefore utilized this model to test whether reducing *COUP-TFII* dosage ameliorates disease progression. The *COUP-TFII* floxed and *Myh6-MerCreMer* alleles were bred into the *CnTg* background to allow genetic removal of one copy of the *COUP-TFII* gene in cardiomyocytes in 7-week-old animals through tamoxifen-induced, cre recombinase-mediated gene excision (Fig. 6a). The majority of mice carrying the *calcineurin* transgene in a *COUP-TFII*^*flox/+*^ background (*CnTg; COUP-TFII*^*flox/+*^*, CnTg/F*^*+*^) exhibit an accelerated rate of sudden death after 12 weeks of age, when compared with mice of CTRL groups *COUP-TFII*^*flox/+*^ (*F*^*+*^) and *Myh6-MerCreMer; COUP-TFII*^*flox/+*^ (*Cre/F*^*+*^) shown in Fig. 6b. Removing one copy of the COUP-TFII gene in cardiomyocytes in the *calcineurin* transgenic background (*CnTg; Myh6-MerCreMer; COUP-TFII*^*flox/+*^*, CnTg/Cre/F*^*+*^) substantially reduces COUP-TFII protein levels (Supplementary Fig. 5a) and leads to an increase in overall survival rate when compared with the CnTg/F^+^ group over a 20-week period (Fig. 6b). This demonstrates that lowering *COUP-TFII* dosage prolongs survival under intense cardiac stress.

Echocardiography imaging before the removal of one copy of *COUP-TFII* (6 weeks old) and post-removal (12 weeks old) was performed to examine structural and functional alterations of the heart. The choice of the 12-week time point is based on previous observations that *CnTg* mice exhibited DCM by 12 weeks^25^, and that the transient cardiac effect exerted by tamoxifen diminished 1 month after treatment^26^. Mice carrying the *calcineurin* transgene already developed eccentric hypertrophy at 6 weeks, before the treatment (Supplementary Fig. 5b,c), consistent with previous findings^25^. Both *CnTg/F*^*+*^ and *CnTg/Cre/F*^*+*^ mice exhibit thicker left ventricular walls (Supplementary Fig. 5b) and increased left ventricular interior dimensions (Supplementary Fig. 5c), when compared with corresponding *F*^*+*^ and *Cre/F*^*+*^ CTRL groups, respectively. At 12 weeks of age, the *CnTg/F*^*+*^ mice develop DCM, exhibiting a greatly increased inner chamber size (Fig. 6c) and reduced RWT (Fig. 6d), compared with CTRL groups of the same age or within the group at age of 6 weeks (Fig. 6c,d and Supplementary Fig. 5c). In contrast, removal of an allele of *COUP-TFII* in *CnTg/Cre/F*^*+*^ mice partially suppresses the dilation (Fig. 6c), and they maintain RWT levels similar to that of 6-week-old mice (Fig. 6d). This result suggests that hearts of *calcineurin* transgenic mice in which one copy of *COUP-TFII* is removed are still able to compensate for stress via a hypertrophic response rather than progressing towards DCM. However, removal of just one copy of *COUP-TFII* failed to rescue the declining contractility (Supplementary Fig. 5d). Taken together, our results indicate that reducing *COUP-TFII* dosage is able to improve survival rates in stressed hearts by partially attenuating the disease progression towards DCM.

## Discussion

Hearts of DCM exhibit mitochondrial dysfunction^4^,^27^, which are proposed to increase oxidative stress, compromise high-energy phosphate production and eventually lead to contractile dysfunction^1^. Normally, oxidative phosphorylation produces ROS as a by-product, which are removed by endogenous antioxidants in healthy mitochondria. However, dysfunctional mitochondria may generate excessive ROS to trigger and augment cellular oxidative stress, and lead to pathogenesis of heart failure^6^,^28^,^29^. In the present study, we found that adult hearts with elevated COUP-TFII levels produce excessive ROS in the mitochondria. The excessive ROS inflict damages on mitochondria, as evidenced by structural abnormality, decreased activities of oxidation-sensitive aconitase and reduced OCRs, eventually leading to contractility reduction and chamber dilation. Our findings are in line with previous studies that demonstrate the key role of excessive mitochondrial ROS production in the development of cardiac dysfunction. For example, increasing ROS levels by knocking out *Sod2*, a principal ROS scavenger located in mitochondrial matrix, leads to heart failure^30^. Conversely, normalizing mitochondrial ROS levels by administering synthetic Szeto-Schiller peptide SS-31 or mitochondrial targeted catalase in hearts ameliorates stress-induced cardiac dysfunction^31^,^32^. Thus, our study links increased COUP-TFII expression, as observed in a subset of DCM patients, to excessive ROS production in adult hearts.

Functioning as a transcription factor, COUP-TFII regulates multiple genes that control mitochondrial ROS levels. COUP-TFII represses expression of many ETC genes in both direct and indirect manners, resulting in decreased activities of ETC enzyme complexes. Modifications of ETC complexes have been shown to disturb electron transportation along the ETC and divert electrons to oxygen for excess ROS production^6^,^33^,^34^. Therefore, our findings suggest that COUP-TFII-mediated ETC defects promote ROS production. At the same time, COUP-TFII overexpression also compromises the endogenous antioxidant capacity by directly repressing the *Sod2* gene. Collectively, the increased oxidative stress observed in COUP-TFII overexpression hearts is likely the result of the combined effect of increased ROS production and decreased ROS detoxification.

Mitochondrial dynamics emerge as an important mechanism in maintaining normal cardiac function by revitalizing mitochondria^35^. The phenotypic resemblance between COUP-TFII OE and *Mfn1/Mfn2* DKO mice^8^ suggests that mitochondrial fusion defects may be an important player in COUP-TFII-induced heart failure. The faster development of aberrant mitochondrial morphology in COUP-TFII OE as compared with *Mfn1/Mfn2* DKO mice (16 days versus 3 weeks, respectively) further suggests that other players may also contribute to our phenotype. The reduced expression of mitochondrial inner membrane fusion gene *Opa1* in the OE hearts could be part of the explanation, as *Opa1* deficiency confers a mitochondrial dysfunction phenotype as strong as the combined deficiency of *Mfn1* and *Mfn2* in MEF cells^36^. In addition, increased COUP-TFII may impair mitochondrial quality control that depends on the Pink1-Mfn2 pathway^24^,^37^. Previous studies show that loss of *Pink1* or *Mfn2* results in mitochondrial dysfunction, increased ROS production and cardiomyopathy^24^,^38^,^39^. Thus, the reduced levels of *Pink1* and *Mfn2* in the OE hearts might lead to pathological accumulation of damaged mitochondria that further contribute to the increase of ROS production.

Transcriptional regulation of mitochondrial ETC genes by the *PGC-1* network has been previously reported^10^,^11^,^12^,^13^ and our results place COUP-TFII upstream of this network. Importantly, we now show that COUP-TFII can directly modulate expression of key ETC genes, indicating that COUP-TFII utilizes two layers of regulating mechanisms to control the essential machinery for oxidative phosphorylation. These COUP-TFII-dependent regulatory mechanisms find further support in the analyses of human specimens, in which COUP-TFII activities inversely correlate with mRNA levels of multiple ETC genes as well as *ERRα, ERRγ* and *PPARα*, whereas failing hearts exhibit higher COUP-TFII and lower *PGC-1/ERR* activities^40^. Notably, *PGC-1α* has been shown to serve as an upstream regulator in promoting *Sod2* expression^41^, and is negatively regulated by COUP-TFII in the hearts. These findings suggest that COUP-TFII may also compromise the ROS detoxifying system through suppressing the *PGC-1α-Sod2* pathway.

Impaired cardiac energy homeostasis is a salient feature of heart failure^42^. In response to a variety of stresses, hearts switch fuel substrate preference from fatty acids to carbohydrates and its proposed mechanism is alteration of expression of cardiac metabolic genes^43^,^44^. Emerging evidence reveals that failing hearts suffer impairment in this substrate selection flexibility^1^,^45^,^46^,^47^. In our mouse model, increased COUP-TFII reduced fatty acid oxidation and reduced glucose usage. This phenotype may be attributed to repressed expression of the *PGC-1* network genes and key glucose utilization genes *Glut4, Hk2* and *Pfkm* by COUP-TFII. Collectively, our COUP-TFII OE mice exhibit a cardiac metabolic phenotype that resembles loss of flexibility of substrate selection in advanced DCM. We propose that alteration of expression of key genes for both fatty acid and glucose metabolism by COUP-TFII underlies the development of the metabolic inflexibility.

In summary, our data indicate that overexpression of COUP-TFII in hearts results in defects in ETC, accumulation of damaged mitochondria and reduced mitochondrial ROS scavenging capacity. All these defects result in the accumulation of oxidative stress and eventually lead to heart failure. Importantly, alterations of gene expression are already observed at day 4 when OE and CTRL hearts are structurally and functionally similar to each other. Therefore, the observable phenotypes are due to COUP-TFII, not secondary to heart failure.

## Methods

### Animals

Mice carried *CAG-S-COUP-TFII* allele and *COUP-TFII flox* allele were described previously^14^,^48^. The cardiomyocyte-specific, tamoxifen inducible cre driver *Myh6-MerCreMer* (*Myh6-MCM*)^19^ and *calcineurin* transgenic mice *Tg(Myh6-Ppp3ca)*^25^ were acquired from the Jackson Laboratory (Stock number 009075). All animals used here are male and are between 2 and 4 months old unless otherwise indicated. All animal experiments adhered to the Guidelines of the Institutional Animal Care and Use Committee of the Baylor College of Medicine and were conducted within the scope of approved animal protocols.

### qRT–PCR

Reverse transcription reactions were carried out using the Transcriptor First Strand cDNA Synthesis kit (Roche, 04379012001) to make cDNA according to the manufacturer’s guide. Quantitative PCR (qPCR) analysis was performed on Applied Biosystems StepOnePlus using the FastStart Universal SYBR Green Master Mix (Roche, 04913850001) or the Taqman Universal master Mix II (Invitrogen, 4440040). Each cycle of the qPCR consists of 95 °C, 15 s, for denaturing and 60 °C, 60 s, for annealing and extension with total 40 cycles performed. Primer sequences are included in Supplementary Table 1.

### Antibodies

Anti-COUP-TFII antibodies are purchased from Perseus Proteomics (H7147, 1:2,000 dilution) or from Cell Signaling (#6434, 1:1,000 dilution). The Pink1 antibody is from Abcam (ab23707, 1:1,000 dilution). The Mfn1 antibody is from Abcam (ab57602, 1:500 dilution). The Mfn2 antibody is from Abcam (ab56889, 1:500 dilution). The Hsp60 antibody is from BD Transduction (#611563, 1:5,000 dilution).

### Cell line

C2C12 (ATCC CRL-1772) are acquired from American Type Culture Collection.

### Expression array analysis

Whole ventricles were collected from 2-month-old animals comprising three *Myh6-MCM* and three *Myh6-MCM; CAG-S-COUP-TFII* mice. RNA extraction was performed using Trizol and Qiagen RNeasy according to the manufacturer’s guides. Affymetrix mouse genome 430 2.0 chips were used in this study. The BCM Genomic and RNA Profiling Core Lab performed the probe generation and subsequent microarray hybridization on Affymetrix mouse genome 430 2.0 chips. Acquired raw data were first processed by the GeneSpring GX software using RMA as the summarization algorithm, Quantile for normalization and median of all samples for baseline transformation. The resulting entity list was filtered on expression (50.0–35272.484) in raw data followed by statistical analysis using one-way analysis of variance with corrected *P* value cutoff at 0.05, asymptotic for *P* value computation and Benjamini–Hochberg for multiple testing correction. Calculated against the CTRL atria group, entities from the mutant atria and CTRL ventricles having absolute fold change greater than 1.2 were considered differentially expressed. The entity lists were subsequently annotated using Affymetrix annotation and analysed by Ingenuity Pathways Analysis (Ingenuity Systems). The NCBI accession number for the expression array study reported in this paper is GSE63759.

The publicly available human array data sets GSE1869 and GSE5406 were scored for manifestation of the mouse model-derived COUP-TFII gene signature, using published methods^49^. A gene signature score (also known as a ‘t-score’) was defined for each external profile as the two-sided *t*-statistic comparing, within the profile, the average of the COUP-TFII-induced genes with the average of the COUP-TFII-repressed genes. In this way, the *t*-score contrasted the patterns of the ‘COUP-TFII-induced’ genes against those of the ‘COUP-TFII-repressed’ genes, could be used to derive a single value denoting coordinate expression of the two gene sets. Where multiple probes in GSE5406 referred to the same gene, the probes with the highest variation was taken to represent the gene; genes were then centred to standard deviations from the median across sample profiles. Expression patterns were visualized as heat maps using Java TreeView^50^. We analysed data from non-ischaemic failing hearts primarily because of the scope of the present study. For GSE1869, we used data from 6 ‘nonfailing’ and 13 ‘Nonischemic, Pre-LVAD Patient group’ hearts for analysis. We exclude the ‘Nonischemic, No-LVAD Patient group’ because the expression profile of this group exhibits similar pattern with the nonfailing group^16^. For GSE5406, we used data from 16 ‘human nonfailing LV myocardium’ samples and 86 ‘human failing LV myocardium, Idiopathic’ specimens for analysis.

### Mitochondrial respiration assay

XF24 extracellular flux analyzer from Seahorse Biosciences was used to measure the rates of oxygen consumption. Mitochondria were isolated from appropriate tissue by homogenization in mitochondrial isolation buffer using a Dounce homogenizer (Kontes) filtered through cheesecloth, and centrifuged at 1,500 *g*, then 9,000 *g*. XF24 cartridge was equilibrated with the calibration solution overnight at 37 °C. XF assay buffer (70 mM sucrose, 220 mM mannitol, 10 mM KH_2_PO_4_, 5 mM MgCl_2_, 2 mM HEPES, 1 mM EGTA, 0.2% BSA, 10 mM pyruvate and 5 mM malate) was prepared and pH adjusted to 7.2 on the day of the experiment. XF assay buffer was used to prepare mitochondrial assay reagents, 2.5 mM ADP, 2 μM oligomycin, 4 μM FCCP, 4 μM antimycin (final concentration). All the reagents were loaded in the ports as suggested by Seahorse Biosciences. OCRs were measured for 4 min with 30 s of mixing and expressed as pmol min^−1^.

### ETC activity assay

For enzymatic assays of respiratory chain complexes I–IV, sufficient amount of tissue or cells were collected. Potassium phosphate buffer (25 mM, pH 7.5) was added to a final volume of 300 μl and the samples were sonicated (5 s pulse x4, 60% power) using a Microson XL2000 Ultrasonic Cell Disruptor (Misonix). For experiments on isolated mitochondria, the mitochondria were also disrupted by sonication as described above. The spectrophotometric kinetic assays were performed at 30 °C in a volume of 175 μl using a monochromator microplate reader (Tecan M200). Complex I activity (NADH:ubiquinone oxidoreductase) was determined by measuring oxidation of NADH at 340 nm (using ferricyanide as the electron acceptor) in a reaction mixture of 25 mM potassium phosphate (pH 7.5), 0.2 mM NADH and 1.7 mM potassium ferricyanide. Complex II activity (succinate dehydrogenase) was determined by measuring the reduction of the artificial electron acceptor 2,6-dichlorophenol-indophenol at 600 nm in a reaction mixture of 25 mM potassium phosphate (pH 7.5), 20 mM succinate, 0.5 mM 2,6-dichlorophenol-indophenol, 10 μM rotenone, 2 μg ml^−1^ antimycin A and 2 mM potassium cyanide. Complex III activity (Ubiquinol/cytochrome *c* oxidoreductase) was determined by measuring the reduction of cytochrome *c* at 550 nm in a reaction mixture of 25 mM potassium phosphate (pH 7.5), 35 μM reduced decylubiquinone, 15 μM cytochrome *c*, 10 μM rotenone and 2 mM potassium cyanide. Complex IV activity (cytochrome *c* oxidase) was determined by measuring the oxidation of cytochrome *c* at 550 nm in a reaction mixture of 10 mM potassium phosphate (pH 7.5) and 0.1 mM reduced cytochrome *c*. Citrate synthase activity was determined by measuring the reduction of 5,5’-dithiobis(2-nitrobenzoic acid) at 412 nm, which is coupled to the reduction of acetyl-CoA by citrate synthase in the presence of oxaloacetate. The reaction mixture consists of 10 mM potassium phosphate (pH 7.5), 100 μM 5,5’-dithiobis(2-nitrobenzoic acid), 50 μM acetyl-CoA and 250 μM oxaloacetate. All activities were calculated as nmol min^−1^ per mg protein, and expressed as a percentage of control activity. Experiments were performed on six independent samples for each genotype.

### Aconitase assay

The activity of mitochondrial aconitase was measured on the basis of conversion of citrate into α-ketoglutarate coupled with NADP reduction (Sigma) and was normalized for total protein. Activity was measured in the native state and after ‘reactivation’ by incubating mitochondria in ferrous ammonium sulfate for 5 min before performing the assay.

### Echocardiogram

For transthoracic echocardiography, mice were anaesthetized using 2% isoflurane in 95% O_2_. Body temperature was maintained on a heated platform, and electrocardiogram and temperature were continuously monitored. Cardiac function was assessed using a VisualSonics VeVo 770 Imaging System (VisualSonics) equipped with high-frequency 30 MHz probe.

### Transaortic constriction

The TAC surgery was performed as described^51^ on adult CD1 mice (strain code: 022, Charles River) of 8–10 weeks of age and between 20 and 25 g of weight. Mice were anaesthetized with isoflurane (2–3%, inhalation) in an induction chamber and then intubated with a 20-G intravenous catheter and ventilated with a mouse ventilator (Minivent, Harvard Apparatus, Inc). Anaesthesia was maintained with inhaled isoflurane (1–2%). A longitudinal 5-mm incision of the skin was made with scissors at midline of sternum. The chest cavity was opened by a small incision at the level of the second intercostal space 2–3 mm from the left sternal border. While opening the chest wall, the chest retractor was gently inserted to spread the wound 4–5 mm in width. The transverse portion of the aorta was bluntly dissected with curved forceps. Then, 6-0 silk was brought underneath the transverse aorta between the left common carotid artery and the brachiocephalic trunk. One 26-G needle was placed directly above and parallel to the aorta. The loop was then tied around the aorta and needle, and secured with a second knot. The needle was immediately removed to create a lumen with a fixed stenotic diameter. The chest cavity was closed by 6-0 silk suture. Sham-operated mice underwent similar surgical procedures, including isolation of the aorta, looping of aorta, but without tying of the suture. The pressure load caused by TAC was verified by the pressure gradient across the aortic constriction measured by echocardiography. Only mice with a pressure gradient >35 mm Hg were analysed for cardiac hypertrophy, echocardiography and gene analysis.

### *Ex vivo* cardiac metabolic analyses

Mice were anaesthetized with chloral hydrate (400 mg kg^−1^) and anticoagulated with heparin (50 IU, i.v.). Hearts were rapidly excised, arrested in ice-cold Krebs–Henseleit buffer, and mounted on a Langendorff apparatus. Hearts were then perfused retrogradely at an afterload of 80 cm H_2_O with non-recirculating Krebs–Henseleit buffer containing glucose (5 mmol l^−1^), sodium oleate (0.4 mmol l^−1^) bound to bovine serum albumin (Probumin, EMD Millipore), [9,3-^3^H]oleate (0.2 μCi ml^−1^), [^U-14^C]glucose (0.1 μCi ml^−1^) and insulin (40 μU ml^−1^; Eli Lilly and Company). The Krebs–Henseleit buffer was equilibrated with 95% O_2_–5% CO^2^ and maintained at a constant temperature of 37 °C. After an initial 30 min stabilization, rates of oleate and glucose oxidation were determined by quantitative collection of [^3^H]_2_O and [^14^C]O_2_ from the coronary effluent as previously reported^52^. Myocardial oxygen consumption (MVO_2_; μmol min^−1^) was calculated by measuring the arterio-venous O_2_ content difference with an YSI 5300A biological oxygen monitor.

### ChIP assay

ChIP analysis of fresh cardiac tissues was performed according to a protocol published in the *Nature Protocols*^53^. Chromatin DNA complexes were collected from mouse ventricles. The mouse monoclonal anti-FLAG antibody M2 (Sigma F1804) was used for the ChIP assay. DNA released from the precipitation was subjected to qPCR analysis for quantification of the presence of specific loci. qPCR analysis was performed on Applied Biosystems StepOnePlus using the FastStart Universal SYBR Green Master Mix (Roche, 04913850001). Each cycle of the qPCR consists of 95 °C, 15 s, for denaturing and 60 °C, 60 s, for annealing and extension performed with total 40 cycles. Primer sequences are included in Supplementary Table 1.

### Oxyblot assay

The Oxyblot assay was perform on freshly isolated mitochondria from ventricles by using the Oxyblot protein oxidation kit from Millipore (S7150) according to the manufacturer’s guide.

### Measurement of ATP content

ATP contents of isolated mitochondria were assessed by the ATPlite kit from Perkin Elmer (6016943) according to the manufacturer’s instruction, and normalized with protein contents.

### Statistical tests

Two-tailed *t*-test is used for statistical analyses unless otherwise indicated.

## Additional information

**How to cite this article:** Wu, S.-P. *et al.* Increased COUP-TFII expression in adult hearts induces mitochondrial dysfunction resulting in heart failure. *Nat. Commun.* 6:8245 doi: 10.1038/ncomms9245 (2015).

## Supplementary Material

Supplementary Information Supplementary Figures 1-5 and Supplementary Table 1
