# Supplementary material for: Increased COUP-TFII expression in adult hearts induces mitochondrial dysfunction resulting in heart failure
Source: Nat Commun. 2015 Sep 10;6:8245. doi: 10.1038/ncomms9245 (PMC4568566; doi:10.1038/ncomms9245)
Supplement: Supplementary Information — Supplementary Figures 1-5 and Supplementary Table 1 [file ncomms9245-s1.pdf]

SUPPLEMENTARY INFORMATION
Supplementary Figures

Supplementary Figure 1

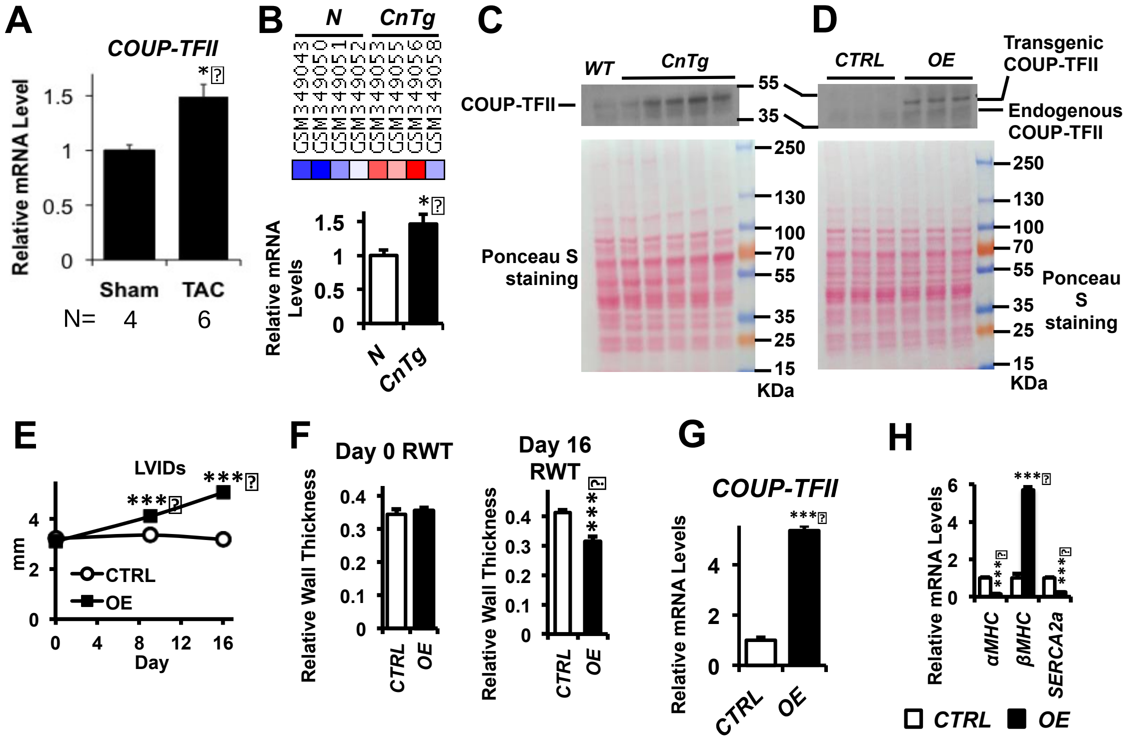

Supplementary Fig. 1. COUP-TFII levels and phenotyping of *COUP-TFII OE* hearts. (A) Ventricular *COUP-TFII* levels by qRT-PCR in 4 sham and 6 TAC mouse hearts 7 days post operation. (B) *COUP-TFII* mRNA Levels in normal (N) and CnTg hearts. Data source: GSE13874. (C) COUP-TFII protein levels by Western analysis of isolated cardiomyocytes from wild type control (WT) and CnTg mice. Ponceau S staining serves as loading control. (D) COUP-TFII protein levels by Western analysis of isolated cardiomyocytes from CTRL and OE mice 9 days post induction of COUP-TFII expression. Transgenic COUP-TFII protein has a higher molecular weight due to fusion with a FLAG-Myc tandem tag. (E, F) Left ventricular interior dimension at systole (LVIDs) and relative wall thickness (RWT) of the same cohort of mice as shown in Fig. 1d &1e. (G, H) Gene expression analysis by qRT-PCR on 3 CTRL and 3 OE ventricles at Day 16. *, $p<0.05$; ***, $p<0.001$ (*t*-Test) between shame and TAC (A), normal and CnTg (B), or CTRL and OE (E, F, G & H). Error bars denote standard error of the mean.

Supplementary Figure 2

Overlapped Gene Signature

	COUP-TFII Induced	COUP-TFII Repressed
ERRα Induced	n.s.	1.16×10^{-6}
ERRα Repressed	0.03	n.s.
PGC-1 Induced	n.s.	3.31×10^{-32}
PGC-1 Repressed	6.62×10^{-4}	n.s.
PPARα^{TAC} Induced	n.s.	2.57×10^{-20}
PPARα^{TAC} Repressed	1.57×10^{-28}	n.s.

SigTerm analysis *p* value

Pathway	Study	Data Source	Induced Genes	Repressed Gene
ERRα	Dufour et al., Cell Metab (2007) 5:345-56. PMID: 17488637.	GSE7196	160	242
PGC-1	Martin et al., Circ Res (2014) 114:626-36. PMID: 24366168.	GSE43798	198	95
PPARα^{TAC}	Smeets et al., Physiol Genomics (2008) 36:15-23. PMID: 18812456.	GSE12337	179	269

Supplementary Fig. 2. Enriched ERRα, PGC-1 and PPARα signatures. For each dataset, derive top genes with $p<0.01$ (one-sided Fisher's exact test), fold>1.4. The PPARα^{TAC} signature was derived from comparative analysis of wild type and Ppara knockout animals under transaortic constriction stress.

Supplementary Figure 3

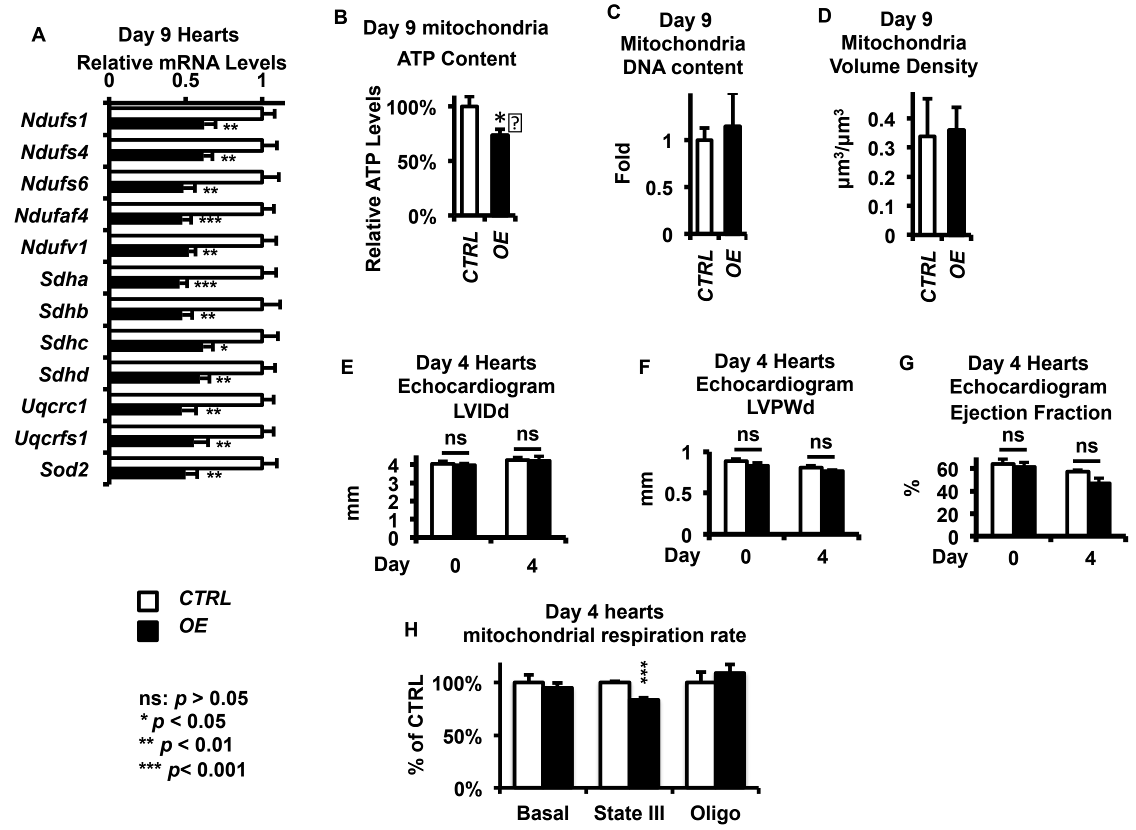

Supplementary Fig. 3. Characterization of Day 4 and Day 9 OE and CTRL hearts. (A) Relative mRNA levels of key ETC and ROS scavenger genes in Day 9 hearts. N=5 for each genotypes. (B) ATP content in mitochondria isolated from ventricles. N = 6 for each genotypes. (C) Mitochondria DNA content of 11 CTRL and 10 OE Day 9 ventricles. (D) Mitochondria volume density calculated from TEM studies on Day 9 ventricles. N= 63 (CTRL) and 62 (OE) fields. Three ventricles of each genotype were examined by transmission electron microscopy. (E-G) Echcardiogram of Day 4 hearts. N = 6 for each genotype. LVIDd, left ventricular interior dimension at diastole; LVPWd, left ventricle posterior wall thickness. (H) Respiration rates of mitochondria isolated from Day 4 hearts. N = 4 for each genotypes. *, $p<0.05$; **, $p<0.01$; ***, $p< 0.001$, ns, $p>0.05$ (*t*-Test) between CTRL and OE. Error bars denote standard error of the mean.

Supplementary Figure 4

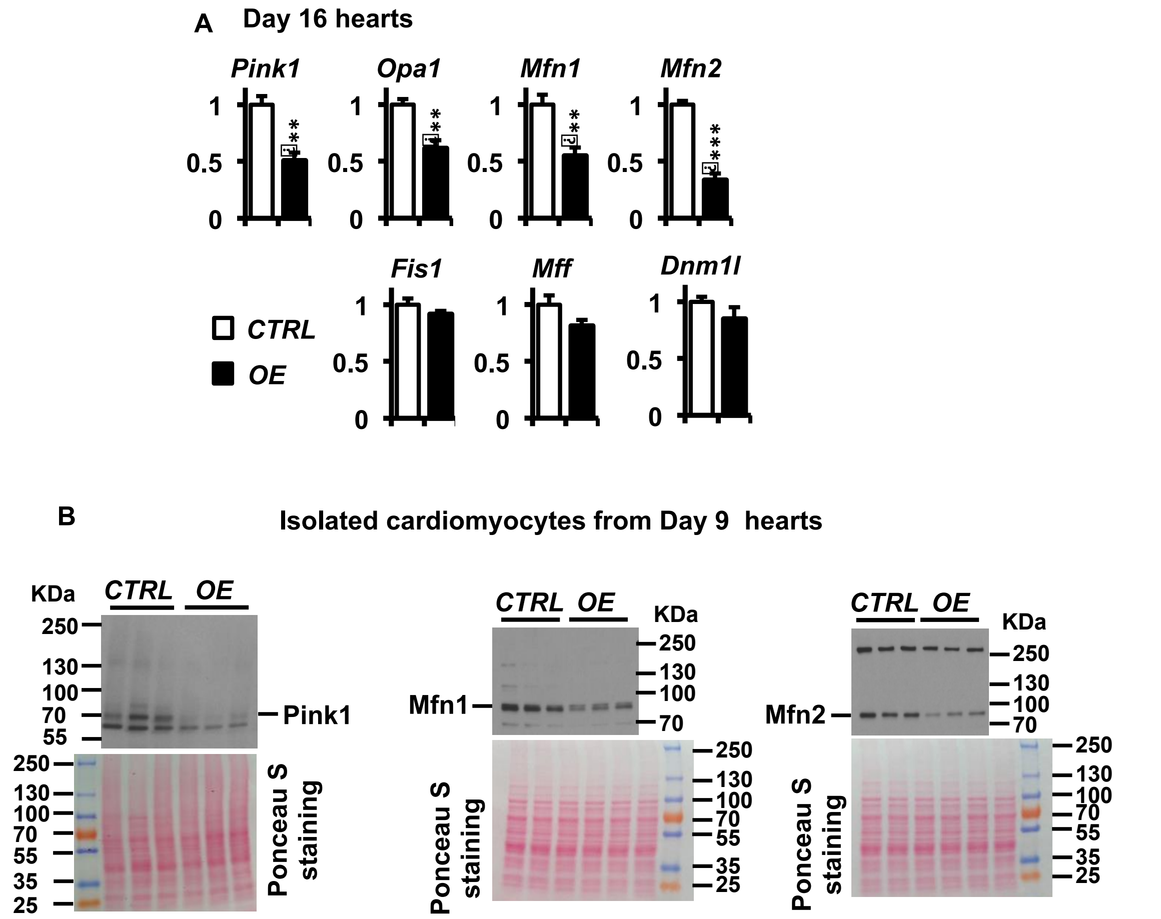

Supplementary Fig. 4. Expression levels of mitochondrial dynamic genes. (A) Relative mRNA levels of major mitochondria dynamics genes in Day 16 ventricles. N=4 for each genotypes. (B) Protein levels of Pink1, Mfn1 and Mfn2 in Day 9 isolated cardiomyocytes. Ponceau S staining serves as loading control. *, p<0.05; **, p<0.01; ***, p< 0.001 (t-Test) between CTRL and OE. Error bars denote standard error of the mean.

Supplementary Figure 5

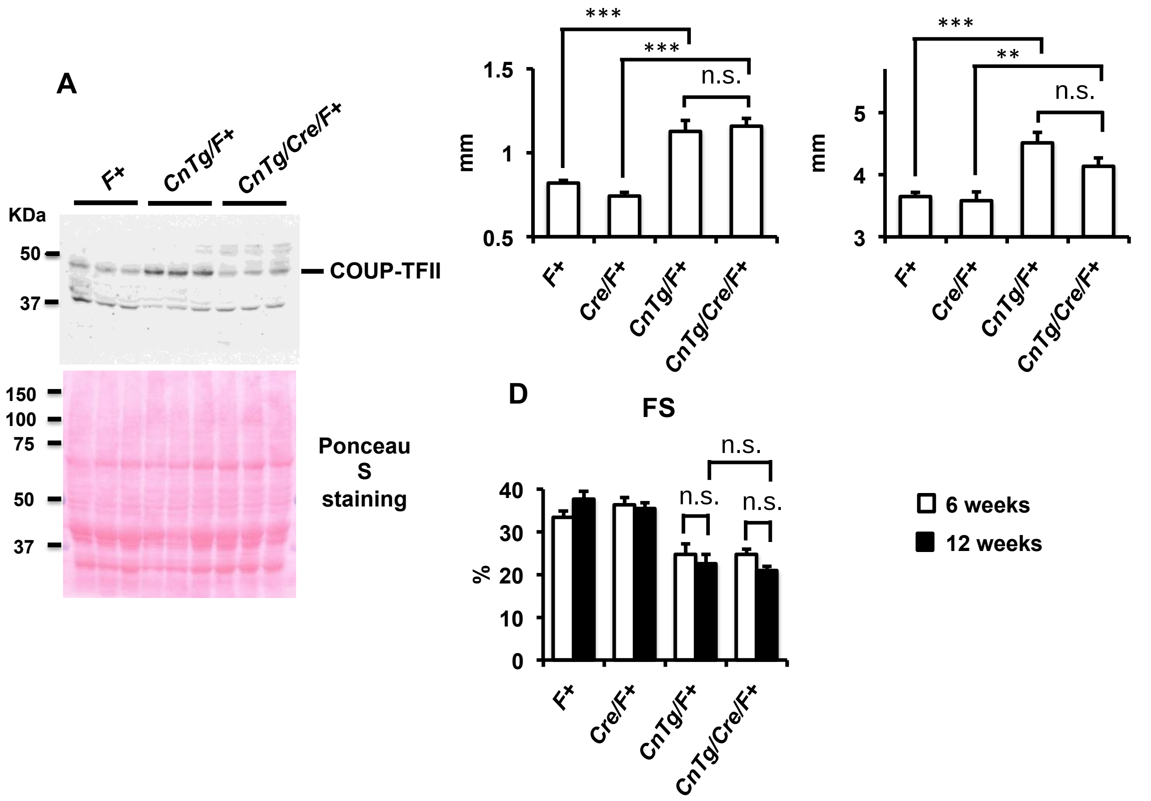

Supplementary Fig. 5. COUP-TFII levels and echocardiogram data of COUP-TFII haploinsufficient mice. (A) Western blot for COUP-TFII protein levels in whole ventricles of 12 week old mice and additional cardiac parameters at 6 and 12 weeks old in denoted genotypes. Ponceau S staining serves as loading control. (B-D) Ecocardiogram of mice in denoted genotypes at 6 weeks old. LVPWd, Left ventricular posterior wall thickness at diastole; LVIDd, Left ventricular interior dimension at diastole; FS, Fractional shortening; **, $p < 0.01$; ***, $p < 0.001$; n.s., $p > 0.05$ (*t*-Test) between denoted groups. Error bars denote standard error of the mean.

Supplementary Table 1. Primers for ChIP-qPCR and qRT-PCR.

ChIP-qPCR

Target	Primer	Sequence
Ndufs1	Forward	cggaccttgacctttaacctc
Ndufs1	Reverse	gcgtccggaaaactccttat
Ndufs6	Forward	caaggtgagcccagtagtgc
Ndufs6	Reverse	acccaagactgacggtagca
Ndufaf4	Forward	gtcctcggcgttttaccg
Ndufaf4	Reverse	cgcttctccacgttgaagtt
Sdha	Forward	taactcaccgccccagtta
Sdha	Reverse	aatcttccaagcccgacac
Sod2	Forward	gaggggccctgattactcc
Sod2	Reverse	gaaaccctggagactttcctc
Mfn2	Forward	atcacagggctgagacatca
Mfn2	Reverse	gactgaaggctgggaggtg
Opa1	Forward	ccccgttatgtcaggttctg
Opa1	Reverse	cgggaagtagtgatgggtca
Pink1	Forward	tcactggtcctggttaagatga
Pink1	Reverse	ctggatcggtgactgataacaa
Ppargc1b	Forward	tgaccttgacaatgttgcttg
Ppargc1b	Reverse	gaacggagcttactggatcg
Esrra	Forward	atgcaagtagccctggtttg
Esrra	Reverse	agttttgtccgctcgtgtct
Esrrg	Forward	aaggtcacattcctgccatc
Esrrg	Reverse	ggcaaggaaaaacagaaggag
Ppara	Forward	ctccagggtctcagttttgc
Ppara	Reverse	gctcgccttttcttgcac

qRT-PCR

Target	Primer	Sequence
aMHC	Forward	acggtgaccataaaggagga
aMHC	Reverse	tgtcctcgatcttgtcgaac
bMHC	Forward	gccctttgacctcaagaaag
bMHC	Reverse	cttcacagtcaccgtcttgc
Rpl32	Forward	ttaagcgaaactggcggaaac
Rpl32	Reverse	ttgttgctcccataaccgatg
Atp2a2	Forward	tcgaccagtcaattcttacagg
Atp2a2	Reverse	cagggacagggtcagtatgc
Ndufs1	Forward	ctgacaacttatgcactgaagagat
Ndufs1	Reverse	ttgagaagataattggaacgtaagtc
Ndufs4	Forward	gatgggaaaatcctttgatgg
Ndufs4	Reverse	gaaggtcagaaccatgttgga
Ndufs6	Forward	ggggaaaagatcacgcatacc

Ndufs6	Reverse	caaaacgaaccctcctgtagtc
Ndufaf4	Forward	caccggagtcagtatccagaa
Ndufaf4	Reverse	ggttcaacttttaccggcaagg
Ndufv1	Forward	cactggtgcaggctcagac
Ndufv1	Reverse	ggctttcacaatgtctgtcg
Sdha	Forward	tgttcagttccaccccaca
Sdha	Reverse	tctccacgacacccttctg
Sdhb	Forward	ctggtggaacggagacaagt
Sdhb	Reverse	gcgttcctctgtgaagtcgt
Sdhc	Forward	gaagaagaacacgagttcaaacc
Sdhc	Reverse	gtgccataggaagagaccattt
Sdhd	Forward	cctgctctgtggtggactact
Sdhd	Reverse	cccatgaacgtagtcggtaac
Sod2	Forward	tgctctaatcaggacccattg
Sod2	Reverse	gtagtaagcgtgctcccacac
Uqcrfs1	Forward	atgtgaagcgacccttcct
Uqcrfs1	Reverse	atgggaaaaacggacagaag
Uqcrc1	Forward	gactacctcaacagacattacaaagc
Uqcrc1	Reverse	gtttctgggcaaggtcca
Mfn2	Forward	cgaggctctggattcacttc
Mfn2	Reverse	caaccagccagctttattcc
Mfn1	Forward	gtgagcttcaccagtgcaaa
Mfn1	Reverse	cacagtcgagcaaaagtagtgg
Opa1	Forward	accaggagaagtagactgtgtcaa
Opa1	Reverse	tcttcaaataaacgcagaggtg
Park2	Forward	gcccggtgaccatgatag
Park2	Reverse	gtgtcagaatcgacctccact
Cd36	Forward	ttgtacctatactgtggctaaatgaga
Cd36	Reverse	cttgtgttttgaacatttctgctt
Acsl1	Forward	ccaaaccagccctatgagtg
Acsl1	Reverse	cttgaaccccttctggatca
Slc27a1	Forward	aaggttcttgcatcctatgctc
Slc27a1	Reverse	tggatcttgaaggtgcctgt
Fabp3	Forward	ctttgtcggtacctggaagc
Fabp3	Reverse	tggtcatgctagccacctg
Cpt1b	Forward	ctcctttcctggctgaggt
Cpt1b	Reverse	gatctggaactgggggatct
Cpt2	Forward	ccaaagaagcagcgatgg
Cpt2	Reverse	tagagctcaggcagggtga
Mlycd	Forward	tcgggaccttcctcataaag
Mlycd	Reverse	ataggcgacaggcttgaaaa
Acadm	Forward	tgtcgaacacaacactcgaaa
Acadm	Reverse	ctgctgttccgtcaactcaa

Acadl	Forward	tggggacttgctctcaaca
Acadl	Reverse	ggcctgtgcaattggagta
Acadvl	Forward	gatcaggtgttcccataccc
Acadvl	Reverse	cccaccagctctttgagaaa
Acaa2	Forward	aaatgtgcgcttcggaac
Acaa2	Reverse	cgttaatcctgcccacaaag
Slc2a4	Forward	gacggacactccatctgttg
Slc2a4	Reverse	gccacgatggagacatagc
HK2	Forward	caactccggatgggacag
HK2	Reverse	cacacggaagttggttcctc
Pfkm	Forward	agatcgtagacgccatcacc
Pfkm	Reverse	ggcccatcacttctaacacaa
Gpi1	Forward	cctctttataatcgcctccaag
Gpi1	Reverse	accactcctttgctgtctctg
Aldoa	Forward	ccaatggcgagacaactacc
Aldoa	Reverse	tatactgggcacagcgttca
Tpi1	Forward	aaaccaaggtcatcgcaga
Tpi1	Reverse	cccggagcttctcgtgta
Gapdh	Forward	tgtccgtcgtggatctgac
Gapdh	Reverse	cctgcttcaccaccttcttg
Pgk1	Forward	tacctgctggctggatgg
Pgk1	Reverse	cacagcctcggcatatttct
Eno1	Forward	aggcgcttagtgctgctc
Eno1	Reverse	atagacatggcgaatttctgg
Pdk4	Forward	cgcttagtgaacactccttcg
Pdk4	Reverse	cttctgggctcttctcatgg
Ppargc1a	Forward	gaaagggccaaacagagaga
Ppargc1a	Reverse	gtaaatcacacggcgctctt
Ppargc1b	Forward	gacgtggacgagctttcact
Ppargc1b	Reverse	gagcgtcagagcttgctgtt
Esrra	Forward	acctctggcagtagctggag
Esrra	Reverse	agcagatgcgacaccagag
Esrrg	Forward	gccagccaaaaagccata
Esrrg	Reverse	caggcatggcatagatcttct
Ppara	Forward	tccgagggctctgtcatc
Ppara	Reverse	gggcagctgactgaggaa
Fis1	Forward	ttctgtgtccaagagcacgc
Fis1	Reverse	agacatagtcccgctgttcc
Mff	Forward	aaatgccagtgtgataatgc
Mff	Reverse	ctggtcttgaaaatgaaatg
Dnml1	Forward	aagccctgagccaatccatc
Dnml1	Reverse	ttgggattactgatgaaccg
Pink1	Probe set	Invitrogen Mm0050827_m1
